# Adjuvant Radiochemotherapy with a 23-Month Overall Survival Time in a Patient after a Surgery due to Splenic Hemangiosarcoma Rupture: A Case Report with the Literature Review

**DOI:** 10.1155/2018/8672407

**Published:** 2018-02-11

**Authors:** M. Bilski, D. Surdyka, I. Paśnik, M. Bilska, P. Cisek, P. Korona, J. Szumiło, L. Grzybowska-Szatkowska

**Affiliations:** ^1^Chair and Department of Oncology, Medical University of Lublin, Lublin, Poland; ^2^Radiation Therapy Department, Lublin Oncological Center, Lublin, Poland; ^3^Chair and Department of Clinical Pathomorphology, Medical University of Lublin, Lublin, Poland; ^4^1st Gynecological Oncology and Gynecology Clinic, Independent Public Clinical Hospital No. 1 in Lublin, Lublin, Poland; ^5^Brachytherapy Department, Lublin Oncological Center, Lublin, Poland; ^6^Radiotherapy Department, Lublin Oncological Center, Lublin, Poland

## Abstract

Spleen sarcoma is one of the most rare soft tissue malignancies. The annual incidence is 0.14–0.25/1,000,000 and the average age of diagnosis is 50 to 73 years. The incidence of this cancer has been increasing. Treatment of choice is surgical splenectomy, which rarely gives good results due to the aggressive course of the disease as well as the high potential for metastasis. Overall survival in primary spleen sarcomas as described by various authors is between 4 and 14 months. 80% of patients after spleen rupture do not survive 6 months. We report the case of a 42-year-old male diagnosed with spleen angiosarcoma. The patient underwent surgery in an emergency mode because of rapid rupture of the organ. Due to positive surgical margins, he underwent adjuvant radiochemotherapy followed by chemotherapy. Overall survival time was relatively long (23 months). The international guidelines provide information based on limited data. The role of postoperative radiotherapy in angiosarcomas remains controversial. Postoperative radiotherapy may increase local disease control, especially after nonradical operation, but this does not translate into improvement in overall survival time of these patients. The case shows that adjuvant radiotherapy as part of cancer treatment strategy may prolong the overall survival.

## 1. Introduction

Sarcomas are malignant tumors that develop from mesenchymal tissue. Among soft tissue sarcomas, spleen sarcoma (sarcoma lienale) is one of the most rare neoplasms. The annual incidence is 0.14–0.25 cases per million [1], and the average age of diagnosis is 50 to 73 years [2–4]. In recent years, the incidence of this cancer has been increasing. Because of the rare nature of these neoplasms and the short overall survival time of patients, often no more than 6 months after diagnosis [5], there are no established standards of clinical practice. The case report presents the therapeutic treatment in patient with splenic hemangiosarcoma, diagnosed at the time of organ rupture.

## 2. Case Report

**Figure 1:**
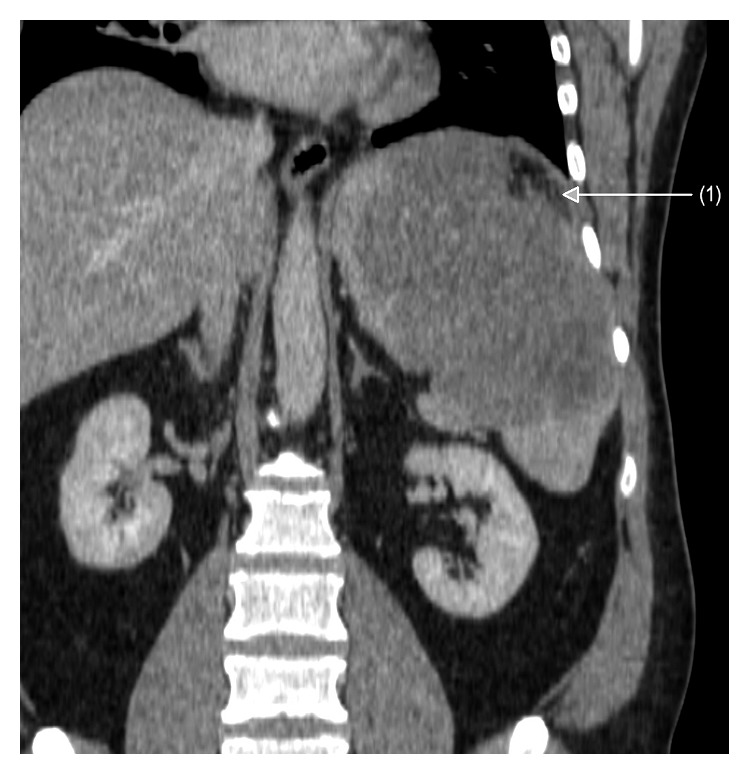
Abdominal CT scan showing an enlarged spleen with its visible rupture (1) and bleeding outside the capsule.

**Figure 2:**
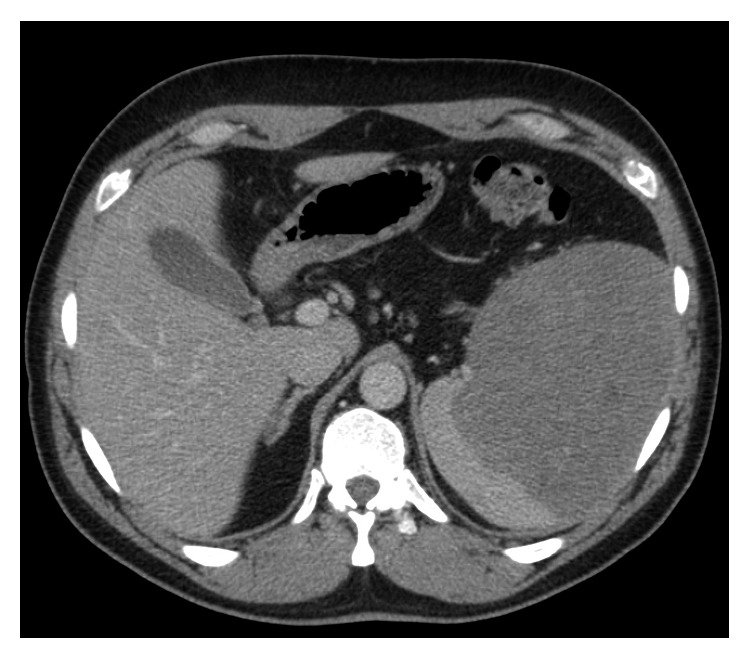
Abdominal CT scan performed before the surgery. Significant spleen enlargement is visible with distinctive area of parenchyma affected by the angiosarcoma.

A 42-year-old patient was hospitalized for severe abdominal pain in the surgery department. That was the first and foremost symptom of the disease reported by him. The following laboratory tests were out of the norm: leukocytes (WBC) 10.83 × 103/mm^3^, hemoglobin (HGB) 10.8 g/dL, red blood cell (RBC) count 4.16 × 106 cells/mm^3^, and C-reactive protein (CRP) −79.25 mg/l. The abdominal spiral computed tomography (CT) examination was performed that showed spleen enlargement with a nonhomogeneous polycyclic contrast-enhanced lesion and bleeding in the infiltrative lesion with slight bleeding outside the fibroelastic capsule of the organ (Figures 1 and 2). The affected spleen adhered to the greater curvature of the stomach, so the invasion of its wall could not have been ruled out. On the 7 December 2011, the spleen was dissected with the larger curvature of the stomach. The postoperative course was uneventful.

**Figure 3:**
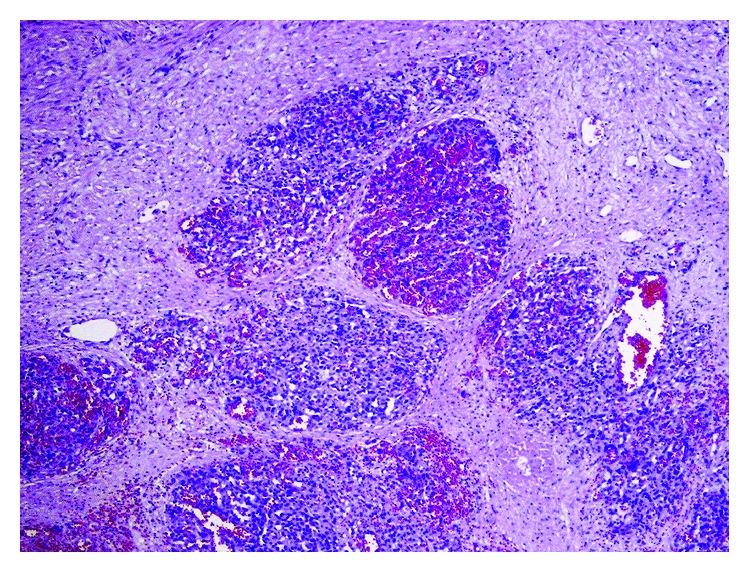
Microscopic view of angiosarcoma with hemorrhagic areas and vascular channels lined by endothelial atypical cells (hematoxylin and eosin staining, 100x).

**Figure 4:**
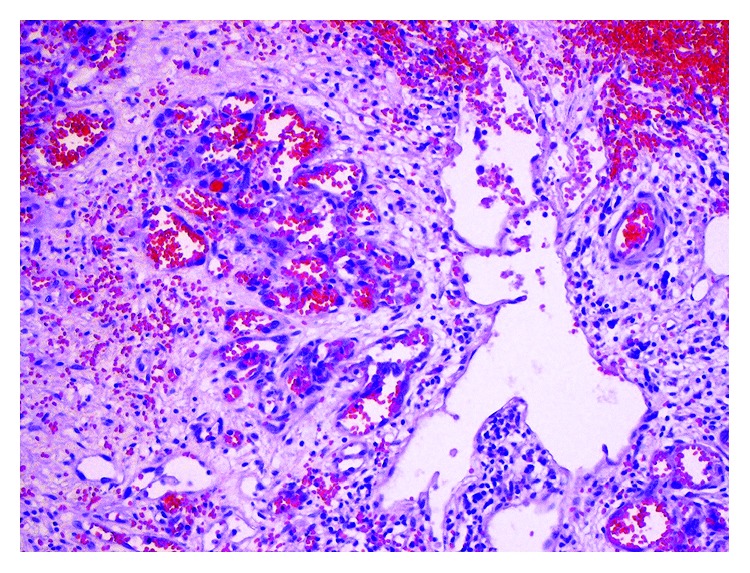
Vascular capillaries of various sizes and cavernous-like spaces lined by endothelial cells with high-grade atypia (hematoxylin and eosin staining, 200x).

**Figure 5:**
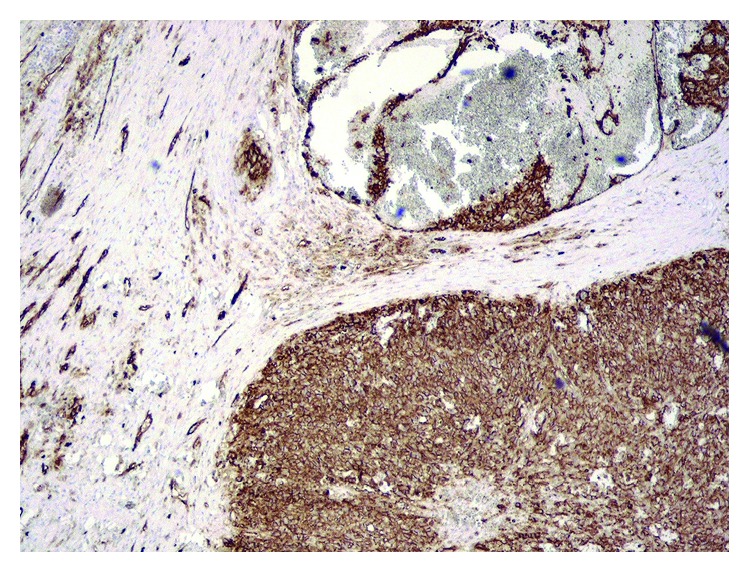
Immunohistochemical stain for CD34 highlights the malignant endothelial cells (200x).

**Figure 6:**
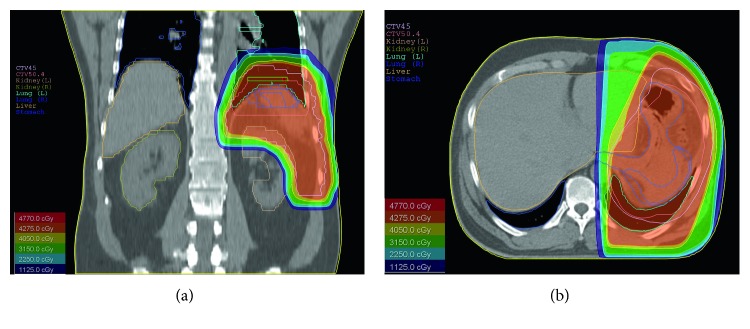
The coronal scope (a) and axial scope (b) of radiotherapy plan and dose distribution illustrated by different isodoses.

Gross examination of surgical specimen showed enlarged spleen (15 × 15 × 7.5 cm) with poorly demarcated, infiltrating tumor (size 3 × 3 × 3 cm) of heterogeneous, hemorrhagic appearance with necrotic areas and cystic spaces filled with blood. Capsule of the spleen was ruptured. On histopathological examination, tumor was composed of nodules formed by vascular capillaries of various sizes and cavernous-like spaces filled with red blood cells. Vascular spaces were lined by endothelial cells with high-grade atypia. Multiple haemorrhages with partly organised thrombi, hemophagocytosis, and hemosiderin deposition were present. Tumor cells showed brisk mitotic activity and high-proliferative index (44% of Ki-67-positive nuclei of neoplastic cells). Immunohistochemically, the neoplastic cells expressed endothelial antigens—CD31 and CD34—smooth muscle actin (ASMA), and vimentin. Focal expression of factor VIII was also present. Cytokeratin, CD117, and S100 protein immunohistostaining were negative (Figures 3–5). A separated sample of the surgical margin from the greater curvature of stomach showed angiosarcoma infiltration. Lymph nodes were not involved. According to TNM (2009) classification, tumor was assessed as T2bN0M0. Patient was qualified for adjuvant chemoradiotherapy. Chemotherapy was administered according to the regimen: paclitaxel 80 mg/m^2^/d, on days 1, 8, and 15 and every 28 days. With the second course of chemotherapy, 3D conformal radiation therapy (3DCRT) has been launched, with 18 MV radiation and cone beam computed tomography (CBCT). A dose of 45 Gy (fraction dose 1.8 Gy) was given to the tumor bed with additional boost to the area of larger curvature of the stomach to a total dose of 50.4 Gy due to the positive surgical margins (Figures 6(a) and 6(b)). During radiotherapy, the patient received one course of chemotherapy. The treatment was well tolerated. No acute toxicity was observed. The patient received 6 cycles of chemotherapy in total. After the third cycle of chemotherapy, stabilization of disease (SD) was confirmed in CT. The treatment was complicated by side effects such as pneumonia, which resolved after antibiotic therapy with amoxicillin, grade III leucopenia, and grade IV neutropenia. The patient received granulocyte-colony stimulating factors (G-CSFs). CT performed in December 2012 showed no local recurrence or dissemination.

The abdominal magnetic resonance imaging in February 2013 showed the presence of multiple small metastatic changes in the liver. Time to dissemination after completion of the first-line treatment was 13 months. The patient was qualified for the second-line chemotherapy consisting of ifosfamide 5 g/m^2^ i.v. on day 1, cisplatin 100 mg/m^2^ i.v. on day 2, and Adriamycin 60 mg/m^2^ i.v. on day 2, every 21 days. G-CSFs were used as prophylaxis of neutropenia. After the third cycle, the patient had pancytopenia. Laboratory examination showed grade II leucopenia (WBC 1.12 × 3.3/mm^3^), grade IV neutropenia (neutrophils 0.23 × 103/mm^3^), grade III anemia (HGB 8.2 g/dl), and grade II thrombocytopenia (platelets 40 × 103/mm^3^). The patient received 3 units of red blood cell concentrates, G-CSFs, and prophylactic antibiotic therapy with ceftazidime. SD was confirmed in a CT scan. The patient was qualified for three further cycles of chemotherapy. Second-line systemic treatment consisting of 6 courses ended in September 2013. Control CT scan showed progression of the disease. There was a confirmed enlargement of liver metastasis and multiple metastases to both lungs. The patient was qualified for palliative 3rd-line monochemotherapy regimen with the Nevelbin 30 mg/m^2^, on days 1, 8, and 15 and every 28 days. After the second cycle of chemotherapy, the patient was hospitalized because of hematuria and anemia: HGB 9.8 g/dl, HCT 29.9%, and D-dimer 59942 ng/ml. In the abdominal ultrasonography, further progression of metastatic changes in the liver and a new hyperechogenic metastasis in the right kidney were demonstrated. Due to a worsening performance status, the patient was qualified for best supportive care (BSC). The patient died in November 2013.

## 3. Discussion

Spleen sarcomas are characterized by a high metastatic potential and lack of specific clinical signs in early stages. There are cases of these neoplasms in the literature, which were accompanied by symptoms of a tumor in the area of left upper abdomen, abdominal pain, fatigue, weight loss, fever, hepatomegaly, splenomegaly, anemia, and thrombocytopenia [3]. In some cases, leucocytosis and thrombocytosis may occur, as in the case of the patient described by us. In about 30% of reported cases, the first manifestation of the disease was a spontaneous rupture of the spleen [4]. Overall survival time in primary spleen sarcomas as described by various authors is between 4 and 14 months. 80% of patients after spleen rupture do not survive 6 months [5]. A slightly better prognosis of up to 11 months was found only among patients who had the spleen removed before rupture [4, 6]. Histopathological diagnosis shows a heterogeneous, nonspecific image. Dominant elements are disorganized vascular connections, which are split by large, atypical endothelial cells distinguished by irregular hyperchromatic nuclei [7]. Both well-differentiated and poorly differentiated changes may have a similar, aggressive course. Mitotic index and tumor size are prognostic factors [8]. The pathologic differential diagnosis included hematoma, infarct, hamartoma, and benign vascular neoplasms (hemangioma and lymphangioma). In our case, other vascular tumor (Kaposi sarcoma) was considered, but extensive necrosis, high-grade atypia of tumor cells, and presence of red blood cells supported the diagnosis of angiosarcoma. Other malignant mesenchymal tumors were ruled out based on typical microscopic features, multiple vascular channel formations, and results of immunohistochemical staining (positive endothelial markers CD31 and CD34). Despite the aggressive course of the disease as well as the high potential for metastasis, splenectomy is still the only method described in the literature that gives the opportunity to achieve satisfactory long-term results. Postoperative assessment of the histopathological sample is fundamental. Negative surgical margins are the most important factors affecting disease-free survival time. In the case of unresectable disease, neoadjuvant treatment in the form of chemotherapy, radiotherapy, or chemoradiotherapy is acceptable, although BSC remains the treatment of choice. In cases of disease progression in the form of metastasis which occurs after the primary surgical treatment, doxorubicin can be used as monotherapy [9]. In the next line of treatment, it is possible to administer paclitaxel monotherapy 1x a week. Thirty patients with metastatic or unresectable, locally advanced disease were included into the phase II prospective study. Two-month PFS was achieved in 74% patients and 4-month PFS in 45%. Average time of progression was 4 months, and overall survival (OS) time was 8 months [10]. Similar results were achieved by Hirata et al. [11]. Interestingly, the expression of TLE3 (transducin-like enhancer of split 3) was associated with a better response to taxanes therapy, which makes it a positive predictive factor [12]. Olaratumab monoclonal antibody binding to the platelet-derived growth factor receptor alpha (PDGFRA) was approved by FDA (U.S. Food and Drug Administration) in October 2016 for the treatment of soft tissue sarcomas in association with doxorubicin [13]. Doxorubicin with olaratumab is currently recommended for the use rather than doxorubicin monotherapy in the first-line treatment of metastatic sarcoma, especially in angiosarcoma. It seems that this regimen should be used instead of chemotherapy based on doxorubicin with ifosfamide because of better tolerance. Some data on the use of angiogenesis inhibitors are promising [14] and even described CR (complete remission) [15] although the addition of bevacizumab to paclitaxel resulted in shortening OS (15.9 versus 19.5 months) [16].

There is little information in the literature about the use of radiotherapy in angiosarcoma, particularly in relation to abdominal or retroperitoneal space origination. In retrospective analysis of 48 patients with different head and neck sarcomas (29.2% angiosarcoma), radiotherapy was used as a separate treatment or part of combined therapy. Five-year LC (local control), LRC (local-regional control), DFS (disease-free survival), and OS values were 87, 73, 63, and 83%, respectively. Patients with angiosarcoma had poorer outcomes than those with other types of sarcomas [17]. Other uses of radiotherapy in patients with angiosarcoma have been reported by Hata et al. In that study, radiotherapy was used in 17 patients with scalp angiosarcomas. The percentage of patients without progression, local relapse, and distant metastases after 1-year follow-up was 86, 67, and 38%, respectively. After 3-year follow-up, it was 86, 38, and 16%, respectively. The authors also point out that a dose of 50 Gy or less may be too low for local control, requiring a dose of at least 70 Gy for bigger lesions [18].

It is important to note that, in most cases, the incidence of angiosarcoma irradiation is extremely rare, with most cases concerning other types of intra-abdominal sarcomas. Because of that, it is important to pay particular attention to the possibility of using irradiation on this type of cancer as adjuvant treatment, especially after a surgical procedure carried out because of a life-threatening situation, such as spleen rupture. In our case, the use of adjuvant chemoradiotherapy after radical surgery (positive microscopic margins) enables to achieve significant prolongation of OS which was 23 months, compared to the average OS of patients with this course of disease, which is only 6 months

## 4. Summary

Treatment results are still very bad in angiosarcoma. Radiotherapy is an important and underestimated treatment for this type of neoplasm, particularly as an adjuvant approach after surgery performed because of splenic rupture. Because of the rarity of the disease, obtaining reliable clinical evidence requires more data.
